# Dillapiole, Isolated from *Peperomia pellucida*, Shows Gastroprotector Activity against Ethanol-Induced Gastric Lesions in Wistar Rats

**DOI:** 10.3390/molecules180911327

**Published:** 2013-09-13

**Authors:** Raúl Rojas-Martínez, Jesús Arrieta, Leticia Cruz-Antonio, Daniel Arrieta-Baez, Antonio Magdiel Velázquez-Méndez, María Elena Sánchez-Mendoza

**Affiliations:** 1 Escuela Superior de Medicina, Instituto Politécnico Nacional, Plan de San Luis y Díaz Mirón, Colonia Santo Tomás, Delegación Miguel Hidalgo, México D.F., 11340, Mexico; E-Mails: romr65@yahoo.com.mx (R.R.-M.); jearrval@yahoo.com.mx (J.A.); 2 Facultad de Estudios Superiores Zaragoza, UNAM, Batalla del 5 de Mayo Esquina Fuerte de Loreto, Ejército de Oriente, México D.F., 09230, Mexico; E-Mail: letycruza@yahoo.com.mx; 3 Centro de Nanociencias y Micro y Nanotecnologías del Instituto Politécnico Nacional, Unidad Profesional Adolfo López Mateos, Luis Enrique Erro S/N, Zacatenco, México D.F., 07738, Mexico; E-Mail: darrieta@ipn.mx; 4 Universidad Tecnológica de la Selva, Entronque Toniná Carretera Ocosingo-Altamirano, Ocosingo, Estado de Chiapas, 29950, Mexico; E-Mail: antoniomagdielv@yahoo.com

**Keywords:** *Peperomia pellucida*, dillapiole, gastroprotection, medicinal plants

## Abstract

*Peperomia pellucida* is a plant used in traditional medicine to treat gastric ulcers. Although this gastroprotective activity was reported, the active compounds have not been identified. Therefore, the aim herein was to identify the most active compound in the gastroprotective activity of *P. pellucida* using an ethanol-induced gastric ulcer experimental rat model. A gastroprotective effect was observed when the hexane and dichloromethane extracts were tested, with the higher effect being obtained with the dichloromethane extract (82.3 ± 5.6%) at 100 mg/kg. Dillapiole was identified as the most active compound in this extract. Although there have been previous reports on dillapiole, this is the first on its gastroprotective activity. Rats treated with this compound at 3, 10, 30 and 100 mg/kg showed 23.1, 56.1, 73.2 and 85.5% gastroprotection, respectively. The effect elicited by dillapiole at 100 mg/kg was not attenuated by pretreatment with indomethacin (10 mg/kg, s.c.), a prostaglandin synthesis blocker, *N^G^*-nitro-l-arginine methyl ester (70 mg/kg, i.p.), a nitric oxide (NO) synthase inhibitor, or *N*-ethylmaleimide (10 mg/kg, s.c.), a blocker of sulfhydryl groups. This suggests that the gastroprotective mechanism of action of dillapiole does not involve prostaglandins, NO or sulfhydryl groups.

## 1. Introduction

Gastric and duodenal ulcers affect a considerable number of people in the world, and according to epidemiological data are becoming increasingly common worldwide [1,2]. This disorder affects approximately 11%–20% of men and 8%–11% of women. In industrialized countries, about 10% of the population suffers from this condition at some time in their life. In North and South America alone, more than two million adults currently have gastric or duodenal ulcers. 

Infection with *Helicobacter pylori* plays an important role in the development of gastric ulcers. Other factors that can lead to this disorder are ethanol, stress, smoking, nutritional deficiencies and frequent ingestion of nonsteroidal anti-inflammatory drugs (NSAIDs) [3]. Gastric ulcers tend to be a chronic disease, which can persist for over 20 years, being characterized by repeated episodes of healing and re-exacerbation [4].

The drugs currently used in the treatment of gastric ulcers are anti-acids, anticholinergics, proton pump inhibitors and H_2_-receptor antagonists. However, there are innumerable adverse effects caused by these allopathic medicines, indicating the need to identify alternatives with fewer side effects. In the search for new drug treatments, metabolites derived from plants used in traditional medicine have provided an important source of alternative therapeutic drugs [5]. 

*Peperomia pellucida* is an annual herb with bright green heart-shaped leaves belonging to the Piperaceae family. It is distributed mainly in Central and South America, Africa, Southeast Asia and Australia, growing in clumps and thriving in the loose, humid soils of tropical to subtropical climates. *P. pellucida* has long been used as a food item as well as a medicinal herb [6]. In folk medicine it is used to prepare infusions and decoctions to treat gastric ulcers as well as gout arthritis, wounds, high blood cholesterol, and skin related problems such as acne [7,8,9]. 

Scientific studies on *P. pellucida* have found anti-inflammatory, analgesic, chemotherapeutic and anti-ulcerogenic properties [6,7,9]. However, the gastroprotector constituents of *P. pellucida* have not yet been identified. Therefore, the aim of the present study was to isolate a gastroprotective constituent from the plant through a bioassay-guided fractionation, using an animal model of gastric lesions induced by absolute ethanol. The role of endogenous NO, sulfhydryl groups and prostaglandins in the gastroprotective effect was evaluated in order to provide information about the mechanism of action of the active compound. The results were compared with the effect of carbenoxolone as the reference drug. 

## 2. Results and Discussion

### 2.1. Bioassay-Guided Fractionation and Isolation of Dillapiole

In recent years, there has been growing interest in alternative therapies, especially the use of natural compounds derived from plants. In this context, extracts of hexane, dichloromethane and methanol from the aerial parts of *P. pellucida* were herein evaluated. The hexane and dichloromethane extracts were found to produce a gastroprotective effect. The dichloromethane extract showed the greatest activity (Table 1), providing the maximum gastroprotective effect (82.3 ± 5.6%) at 100 mg/kg.

**Table 1:** Gastroprotective effect of *P. pellucida* extracts on ethanol-induced ulceration in rats.

Treatment	Dose (mg/kg)	n	UI (mm^2^)	Gastroprotection (%)
Control	---	8	85.4 ± 9.4	---
Hexane extract	30	8	39.1 ± 5.1 *	54.1 ± 6.1
	100	8	36.7 ± 3.2 *	56.9 ± 3.7
Dichloromethane extract	30	8	34.7 ± 5.2 *	59.2 ± 6.1
	100	8	15.0 ± 4.8 *	82.3 ± 5.6
Methanol extract	30	8	69.9 ± 7.9	18.0 ± 7.5
	100	8	81.0 ± 6.2	5.1 ± 5.1
Carbenoxolone	100	8	21.8 ± 3.9 *	72.0 ± 5.0

* *p* < 0.05 *vs.* control group; UI = Ulcer index.

**Figure 1:**
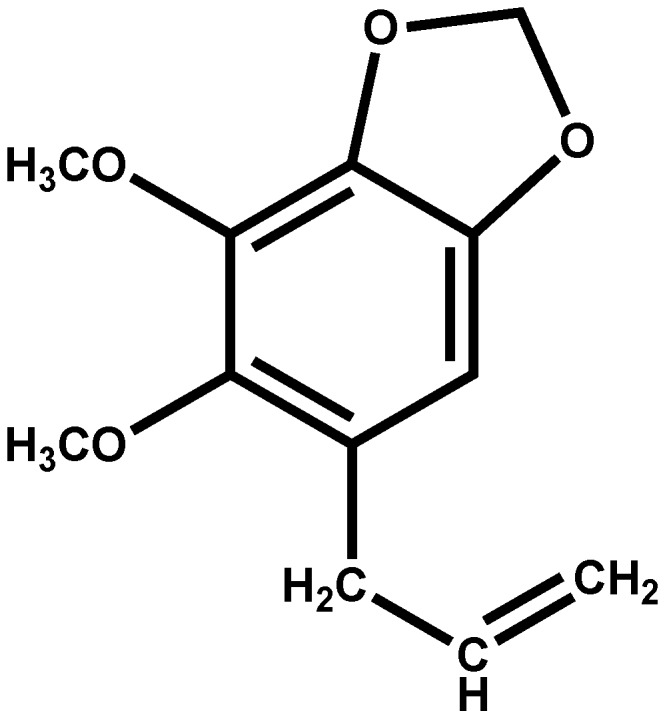
The structure of dillapiole.

Consequently, the dichloromethane extract was separated over a silica gel column, giving five fractions (Table 2). Four of them had considerable gastroprotective activity, which suggests that *P. pellucida* contains more than one active compound. Since F1 was found to be most active fraction, an aliquot was separated on a silica gel column. Elution was performed with hexane and hexane/EtOAc mixtures, obtaining 50 fractions of 20 mL each. Fractions 7–15 (hexane) yielded an essential oil (3.2 g, 12.8%), which was characterized by ^1^H- and ^13^C-NMR spectroscopy. The spectral data was compared to that in the literature [10], and the oil was identified as dillapiole, a polyalkoxybenzene (Figure 1). Dillapiole was previously isolated from *P. pellucida* [11], and it reportedly has insecticidal [12], antimicrobial [13] and anti-inflammatory activity [14]. 

**Table 2:** Gastroprotective effect of the fractions of dichloromethane extract on ethanol-induced ulceration in rats.

Treatment	Dose (mg/kg)	n	UI (mm^2^)	Gastroprotection (%)
Control	---	8	72.0 ± 12.4	---
F1	100	8	9.7 ± 4.3 *	86.4 ± 6.0
F2	100	8	36.5 ± 6.7 *	49.3 ± 9.3
F3	100	8	47.4 ± 7.5 *	48.0 ± 11.2
F4	100	8	27.6 ± 7.2*	61.6 ± 9.6
F5	100	8	42.5 ± 7.2	40.0 ± 10.0

* *p* < 0.05 *vs.* control group; UI = Ulcer index.

**Figure 2:**
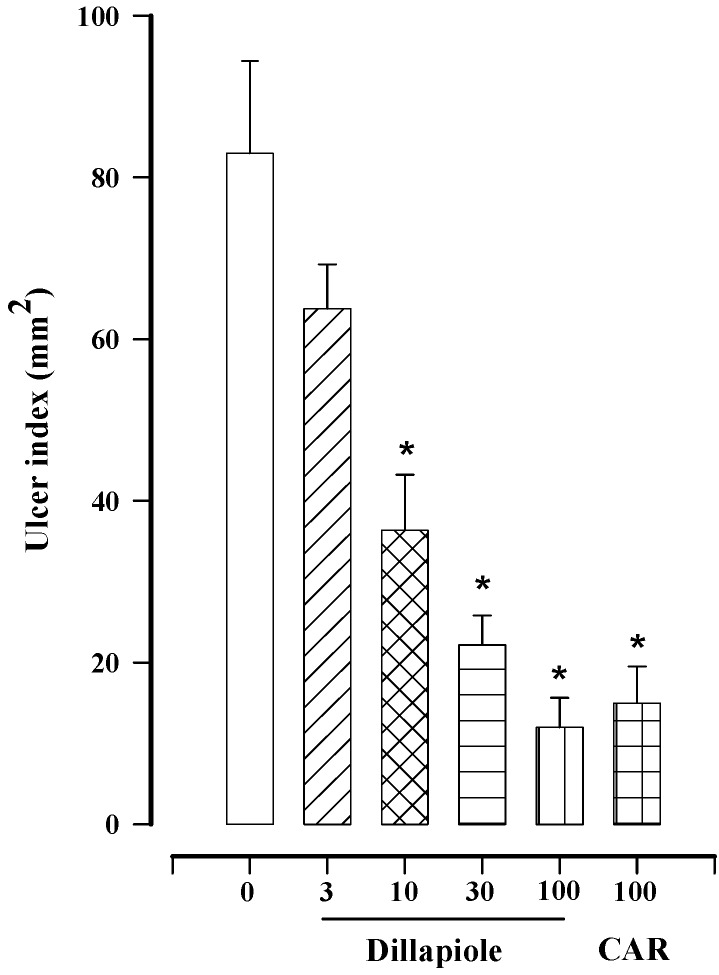
Effect of different doses of dillapiole (3–100 mg/kg) and carbenoxolone (CAR; 100 mg/kg) on gastric lesions induced in rats by absolute ethanol. Bars represent the mean ± SEM (n = 8). * *p* < 0.05 *vs.* the respective control; Dunn’s multiple comparison test after the Kruskal-Wallis test.

We are now reporting for the first time the gastroprotective activity of dillapiole. The effect of this compound was found to be dose dependent, reaching the maximum effect (85.7 ± 4.3%) at a dose of 100 mg/kg (Figure 2), which is the same value obtained with F1 (Table 2). This suggests that the activity of F1 is probably due only to dillapiole. The maximal gastroprotective effect induced by carbenoxolone (reference drug) was 80.7 ± 5.5% at a dose of 100 mg/kg (Figure 2), a value very similar to that obtained with dillapiole at the same dose. Thus, it seems that both compounds have an equivalent efficacy. Although there are compounds in *P. pellucida* other than dillapiole that contribute to its gastroprotective effect, they were not considered in the present study. 

The long term use of NSAIDs, which are known to have an antinociceptive and anti-inflammatory effect [3], often leads to gastric ulcers. All NSAIDs appear to share a common mechanism the inhibition of cyclooxygenase enzymes, leading to a decrease in the synthesis of various prostaglandins and tromboxanes. Great efforts have been made to find selective inhibitors of COX enzymes in order to reduce the side effects of NSAIDs. It is known that cylooxygenase-2 is inducible and its inhibition is responsible only for anti-inflammatory effects. On the other hand, cyclooxygenase 1 is constitutive and thus its inhibition is responsible for anti-inflammatory effects as well as side effects. Consequently, a drug capable of selectively blocking cyclooxygenase-2 should reduce adverse effects on the gastrointestinal tract [15,16]. However, one study reported that a selective inhibitor of cyclooxygenase-2 damages the mucosa [17], while another found a limited effect of cyclooxygenase-2 selective NSAIDs on gastric cyclooxygenase activity [18]. As can be seen, the efforts to find selective NSAIDs have met many unexpected obstacles. 

As mentioned previously, a common route for drug discovery is the extraction of compounds from plants traditionally used for medicinal purposes. Along these lines, it has been previously demonstrated that dillapiole possesses anti-inflammatory effects [14], and in the present study a gastroprotective activity was found too. This combination of activities is interesting, due to the fact that NSAIDs (prescribed to reduce inflammation) and the gastric acid-inhibiting drugs are normally administered in order to counter the risks of gastric damage. Hence, it is possible that this dual activity of dillapiole could avoid the co-administration of two drugs, or reduce the doses of one or more of these. 

### 2.2. Effect of Indomethacin, L-NAME, and NEM on the Gastroprotection of Dillapiole

In an attempt to explain the mechanism of action of dillapiole, we studied the possible involvement of prostaglandins, NO, and sulfhydryl compounds. To determine the possible participation of endogenous prostaglandins in the gastroprotective effect of dillapiole, animals were pretreated with indomethacin (an inhibitor of COX) before the administration of ethanol. It is well known that the inhibition of COX decreases prostaglandin production, leading to gastric hypermotility and microvascular disturbances, which in turn promote the activation and infiltration of neutrophils, ROS production, lipid peroxidation [19], and an increase of gastric acidity [20]. 

**Figure 3:**
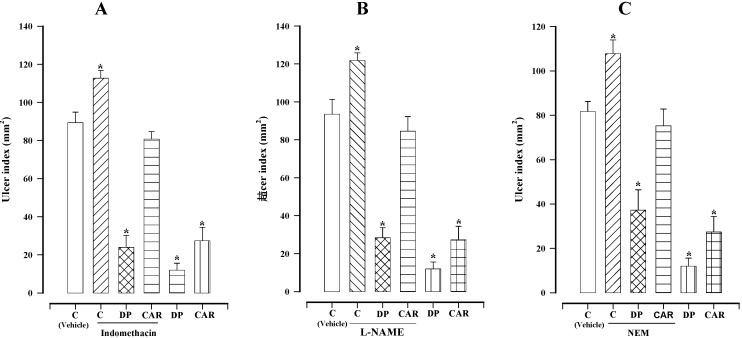
Effect of dillapiole (DP) and carbenoxolone (CAR) at 100 mg/kg on gastric lesions induced by ethanol in rats pretreated with (**A**) indomethacin (10 mg/kg), (**B**) l-NAME (70 mg/kg) or (**C**) NEM (10 mg/kg). Bars represent the mean ± SEM (n = 10). * *p* < 0.05 *vs.* the respective control; Dunn’s multiple comparison test after Kruskal-Wallis test.

When comparing the control group (no treatment) with animals treated only with indomethacin (10 mg/kg), it was observed that this compound increased ethanol-induced gastric lesions (112.8 ± 4.2 mm^2^*versus* 89.7 ± 5.5 mm^2^; Figure 3A). However, pretreatment with indomethacin followed by the oral administration of dillapiole (100 mg/kg) resulted in the same cytoprotection (24.0 ± 6.3 mm^2^) afforded by treatment with dillapiole alone. This result shows that endogenous prostaglandins are not implicated in the action mechanism of dillapiole. Contrarily, prostaglandins do in fact play a role in the mechanism of action of carbenoxolone (Figure 3A).

The mechanism of action of some gastroprotector compounds obtained from medicinal plants involves nitric oxide [21,22]. Furthermore, it is well recognized that NO is involved in the modulation of acid, alkaline and mucus secretion, gastric mucosal blood flow, and the reduction of lipid peroxidation [23,24]. Therefore, we decided to evaluate the possible role of endogenous NO in the gastroprotector activity of dillapiole by pretreating animals with the well-known NOS inhibitor, L-NAME. 

When comparing the control group (no treatment) with animals treated only with L-NAME (70 mg/kg), it was observed that this compound increased ethanol-induced gastric lesions (121.8 ± 4.0 mm^2^*versus* 93.5 ± 7.8 mm^2^; Figure 3B). However, pretreatment with L-NAME followed by the oral administration of dillapiole (100 mg/kg) resulted in the same cytoprotection (28.4 ± 5.2 mm^2^) afforded by treatment with dillapiole alone. This result shows that endogenous NO is not implicated in the gastroprotector mechanism of dillapiole. Contrarily, NO does in fact play a role in the mechanism of action of carbenoxolone (Figure 3B). 

Ethanol-induced gastric damage is associated with a significant decrease in mucosal sulfhydryl compounds [25]. It is known that these compounds bind to the free radicals that form after tissue injury by noxious agents. Moreover, sulfhydryls compounds may protect mucus, because mucus subunits are joined by disulfide bridges, which if reduced render mucus water-soluble [25]. Therefore, we investigated the possible involvement of endogenous sulfhydryl compounds in the gastroprotective effect of dillapiole by pre-treating animals with NEM (a sulfhydryl blocker).

When comparing the control group (no treatment) with animals treated only with NEM (10 mg/kg), it was observed that this compound increased ethanol-induced gastric lesions (107.7 ± 6.1 mm^2^ *versus* 81.7 ± 4.5 mm^2^; Figure 3C). However, pretreatment with NEM followed by the oral administration of dillapiole (100 mg/kg) resulted in the same cytoprotection (37.2 ± 9.1 mm^2^) afforded by treatment with dillapiole alone. This result shows that endogenous sulfhydryl compounds are not implicated in the gastroprotector mechanism of dillapiole. Contrarily, endogenous sulfhydryl compounds do in fact play a role in the mechanism of action of carbenoxolone (Figure 3C). 

Given the fact that a possible role of prostaglandins, NO and sulfhydryl compounds in relation to the mechanism of gastroprotective action of dillapiole could be discounted, other possible mechanisms of action need to be investigated in future studies. 

## 3. Experimental

### 3.1. General Procedures

The ^1^H and ^13^C-NMR spectra were recorded in a CDCl_3_ solution on a BRUKER Advance III Spectrometer (Palo Alto, CA, USA), at 750 MHz. 

### 3.2. Plant Material

The leaves and stems of *P. pellucida* were collected in the Ejido Zaragoza Nueva Alemania, Municipality of Tapachula, the State of Chiapas, Mexico, during August of 2012. The plant was identified and registered by Oscar Farrera Sarmento working in the Herbario Eizi Matuda - Facultad de Ciencias Biológicas. Universidad de Ciencias y Artes de Chiapas of the State of Chiapas, Mexico. A specimen of the original collection can be found with the voucher number 17218.

### 3.3. Extraction and Preliminary Fraction

The leaves and stems of *P. pellucida* were dried at room temperature (22 ± 2 °C) in the shade. After grinding 3.5 kg of the plant material by maceration at room temperature (22 ± 2 °C), extractions were performed three times during 3 days, first with hexane (20 L), then dichloromethane (20 L) and finally methanol (20 L). Evaporation of the solvents in vacuum yielded 60.4, 140.4, and 150 g of syrupy residues, respectively. The dichloromethane extract obtained from the leaves and stems of *P. pellucida* showed the most gastroprotective effect (Table 1). Thus this extract (130 g) was subjected to percolation over a silica gel column by using a step gradient of hexane (2 L, F1) hexane/EtOAc (9:1, 2 L, F2), hexane/EtOAc (7:3, 2 L, F3), hexane/EtOAc (1:1, 2 L, F4) and EtOAc (2 L, F5). A portion of the most active fraction (F1, 25 g) was chromatographed on a silica gel column (500 g) by using a step gradient of hexane, hexane/EtOAc and EtOAc. From this procedure we obtained 50 fractions of 20 mL each. Fractions 7 to 15 (Hexane) yielded an essential oil (3.2 g), which was identified by comparing its spectral data ( ^1^H and ^13^C-NMR) with that of the literature [10] as dillapiole. ^1^H-NMR (750 MHz, CDCl_3_) δ 6.37 (s, 1H), 5.97–5.91 (m, 1H), 5.90 (d, *J* = 9.1 Hz, 2H), 5.10–5.02 (m, 2H), 4.03 (s, 3H), 3.77 (s, 3H), 3.33 (d, *J* = 6.6 Hz, 2H). ^13^C-NMR (189 MHz, CDCl_3_) δ 144.62, 144.32, 137.64, 137.41, 135.95, 126.05, 115.54, 102.75, 101.11, 77.27, 77.10, 76.93, 61.25, 59.93, 33.92. These data match those in the literature [10].

### 3.4. Animals

All the experiments were performed with male Wistar rats, weighing 180–220 g, obtained from the animal house of the Universidad Autónoma Metropolitana, Xochimilco campus, Mexico City, Mexico. Procedures involving animals and their care were conducted in conformity with the Mexican Official Norm for Animal Care and Handling (NOM-062-ZOO-1999), and in compliance with international rules on care and use of laboratory animals. Unless otherwise specified, the rats were placed in single cages with wire-net floors and deprived of food 24 h before experimentation. Animals were allowed free access to tap water throughout the experimental procedures. All experiments were carried out with 8–10 animals per group. 

### 3.5. Drugs and Dosage

Carbenoxolone (Sigma-Aldrich Co. St. Louis, MO, USA), used as the gastroprotective reference drug, was prepared freshly for each use, suspended in 0.5% Tween 80 and administered by the intragastric route. Control rats received the vehicle (0.5% Tween 80) in the same volume (0.5 mL/100 g) and by the same route. *N*-nitro-L-arginine methyl ester (L-NAME), *N*-ethylmaleimide (NEM) and indomethacin (IND) were purchased from Sigma Chemical Co. (St. Louis, MO, USA). 

### 3.6. Acute Gastric Ulcer Induced by Absolute Ethanol

A gastric ulcer was induced by orally administering absolute ethanol (1 mL) [5]. The extracts or drugs were administered to the corresponding group 30 min before ethanol administration. Two hours after ethanol administration, the animals were sacrificed in a CO_2_ chamber. The stomach and duodenum were dissected, inflated with formalin (10 mL), and then placed in 2% formalin for 5 min to fix both the inner and outer layers. The duodenum was opened along its anti-mesenteric side and the stomach along the greater curvature. The damaged area (mm^2^) was measured under a dissection microscope (×10) with an ocular micrometer. The ulcer index was calculated as the sum of all the lesions (area in mm^2^) in the stomach of each animal. Gastroprotection (%) was calculated according to:

% gastroprotection = (UIC − UIT) × 100/UIC
(1)

where UIC and UIT are the ulcer indexes, of the control and test animals, respectively [5]. 

### 3.7. Ethanol-Induced Gastric Mucosal Lesions in Indomethacin Pretreated Rats

To assess the participation of endogenous prostaglandins in the gastroprotective effect of the compounds, a control group received a subcutaneous injection of 5 mM NaHCO_3_ in saline solution and an experimental group an injection of indomethacin (10 mg/kg dissolved in NaHCO_3_ at 5 mM) by the same route [5]. After 75 min, subgroups of animals in each of these two groups received one of three oral treatments (saline solution, 100 mg/kg dillapiole or 100 mg/kg carbenoxolone). Absolute ethanol was given to each rat 30 min after dillapiole or carbenoxolone administration and the rats were sacrificed 2 h later in a CO_2_ chamber. The stomachs were subsequently removed to measure the ulcer index, as aforementioned. 

### 3.8.Ethanol-Induced Gastric Mucosal Lesions in L-NAME Pretreated Rats

To assess the participation of endogenous NO in the gastroprotective effect of the compounds, l-NAME (70 mg/kg dissolved in saline solution) was intraperitoneally injected into 3 experimental groups 30 min before the administration of either the vehicle, dillapiole (100 mg/kg) or carbenoxolone (100 mg/kg) by the oral route [5]. A control group received no treatment. Absolute ethanol was then given to each rat in these four groups 30 min later, and the animals were sacrificed 2 h after the administration of ethanol to measure the ulcer index. 

### 3.9. Ethanol-Induced Gastric Mucosal Lesions in NEM Pretreated Rats

To assess the participation of endogenous sulfhydryls in the protective effects of dillapiole and carbenoxolone, NEM (dissolved in saline solution) was subcutaneously injected (10 mg/kg) in 3 groups of animals 30 min before the oral administration of either the vehicle, dillapiole (100 mg/kg) or carbenoxolone (100 mg/kg) [5]. A control group received no treatment. Absolute ethanol was given to each rat in these four groups 30 min later and rats were sacrificed 2 h after the administration of ethanol to measure the intensity of the gastric ulcer. 

### 3.10. Statistics

Data are presented as the mean ± SEM for 8–10 rats per group. Statistical significance between treatments was evaluated by the Kruskal-Wallis test, followed by Dunn’s multiple comparison tests, with *p* ≤ 0.05 considered as significant.

## 4. Conclusions

In the current study dillapiole was identified as the most active gastroprotective agent of *Peperomia pellucida*. The mechanism of the gastroprotective action shown by dillapiole is not related to endogenous NO, prostaglandins or sulfhydryl groups.

## Supplementary Materials

Supplementary materials (^1^H-NMR and ^13^C-NMR spectra of dillapiole) can be accessed at: http://www.mdpi.com/1420-3049/18/9/11327/s1.
