# Supplementary figures and images for: Dillapiole, Isolated from Peperomia pellucida, Shows Gastroprotector Activity against Ethanol-Induced Gastric Lesions in Wistar Rats
Source: Molecules. 2013 Sep 13;18(9):11327–37. doi: 10.3390/molecules180911327 (PMC6269875; doi:10.3390/molecules180911327)

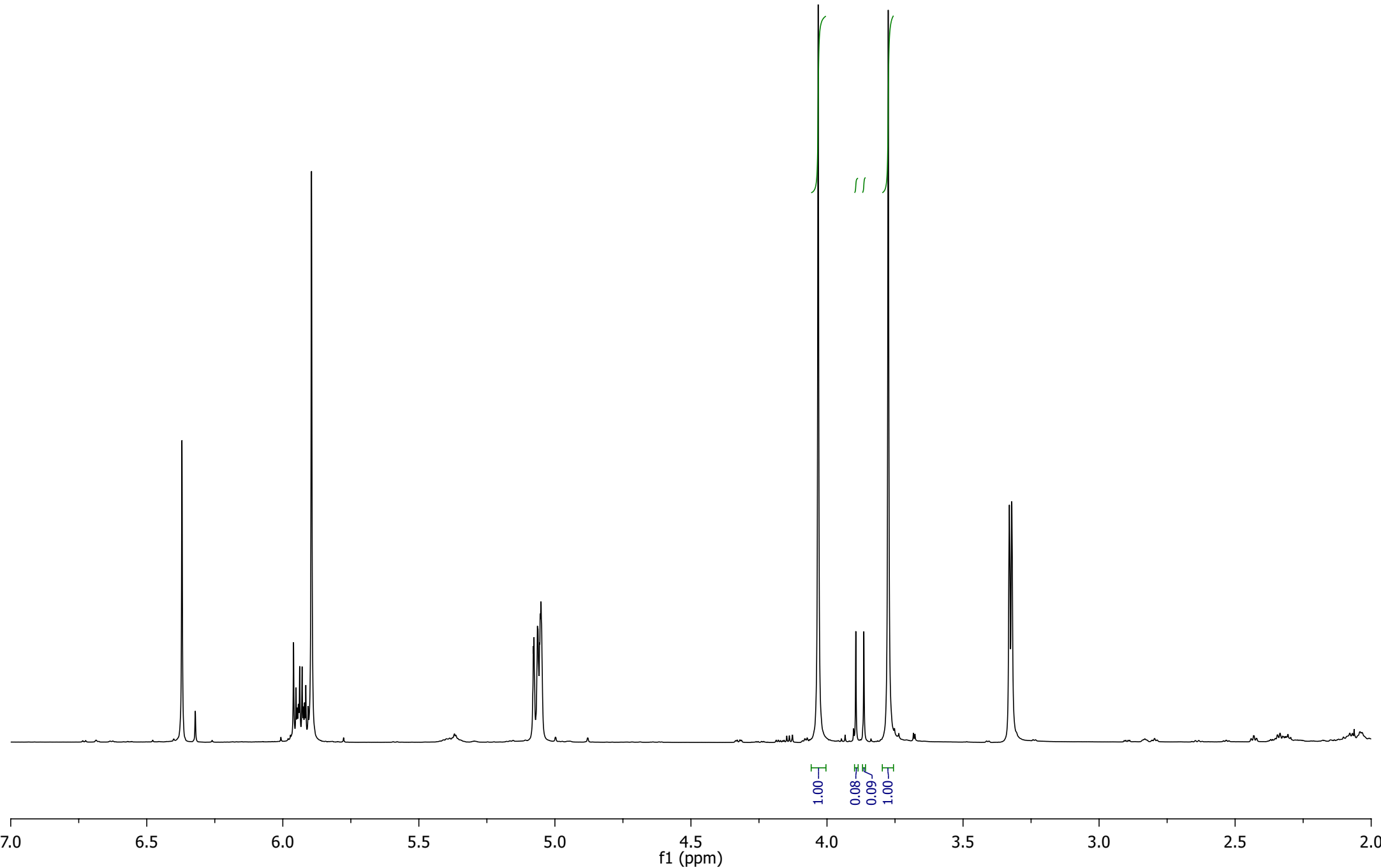

Supplement: Supplementary file 1 [file molecules-18-11327-s001.pdf]
